# NF-κB RelA is a cell-intrinsic metabolic checkpoint restricting glycolysis

**DOI:** 10.1186/s13578-024-01196-7

**Published:** 2024-01-20

**Authors:** Liwen Li, Lei Han, Zhaoxia Qu

**Affiliations:** 1 Department of Microbiology and Molecular Genetics, UPMC Hillman Cancer Center, University of Pittsburgh School of Medicine, Pittsburgh, PA 15213 USA; 2 Department of Molecular Microbiology and Immunology, Hastings Center for Pulmonary Research, Norris Comprehensive Cancer Center, University of Southern California Keck School of Medicine, 1450 Biggy Street, NRT 4506, Los Angeles, CA 90033 USA

**Keywords:** NF-κB, RelA/p65, Metabolic checkpoint, Metabolic switch, Metabolism, Glycolysis, Oxidative phosphorylation, Macrophages

## Abstract

An intrinsic link between metabolism and function in immune cells, and in particular macrophages, has been well established recently. However, the molecular mechanisms controlling the metabolic switch in these sentinel cells for their integral roles in host defense, inflammation, homeostasis, and pathogenesis remain largely unknown. Here, we identify the master transcription factor NF-κB RelA as a vital cell-intrinsic checkpoint restricting aerobic glycolysis to favor mitochondrial oxidative phosphorylation (OXPHOS) and “M2” activation (alternative anti-inflammatory and pro-tumorigenic activation, in contrast to classical pro-inflammatory and anti-tumor M1 activation) of macrophages under oncogenic stress. RelA specific knockdown or genetic deletion in macrophages causes metabolism to shift away from OXPHOS toward glycolysis, resulting in drastically decreased oxygen consumption but significantly increased lactate and ATP production. The metabolic change in RelA deficient cells is associated with the decrease in the expressions of the OXPHOS gene SCO2 as well as the M2 marker and function genes arginase-1 and VEGF. These data suggest that RelA induces SCO2 expression to enhance OXPHOS and restrict glycolysis in macrophages for their pro-tumorigenic activation.

**Supplementary Information:**

The online version contains supplementary material available at 10.1186/s13578-024-01196-7.

## Dear editor,

Macrophages are functionally highly plastic and play central roles in host defense, inflammation, homeostasis, and pathogenesis including lung cancer, the number one cancer killer of both men and women [1–3]. Recent studies have revealed the vital role of metabolism in shaping the phenotype and function of macrophages [1, 4]. Upon activation by pathogens, for example, aerobic glycolysis is induced in macrophages, providing rapid energy and initiating quick pro-inflammatory response to eliminate the invading pathogens. Following pathogen clearance, the metabolism is then switched toward oxidative phosphorylation (OXPHOS), offering long term energy and promoting anti-inflammatory response and wound healing. The glycolytic, pro-inflammatory and oxidative, anti-inflammatory macrophages are often simply called as M1 and M2 macrophages, respectively. Of note, blocking oxidative metabolism in macrophages prevents their M2 polarization and concomitantly drives them into an M1-like state, whereas forcing oxidative metabolism in glycolytic M1 macrophages skews them into an M2-like phenotype [4]. Not surprisingly, the highly adaptive metabolic engine and functional plasticity of macrophages important for host defense and homeostasis are often abused under various pathogenic conditions and cancer in particular. Tumor cells create a microenvironment to preserve OXPHOS and impede glycolysis in associated macrophages (TAMs), repressing M1-like anti-tumor activation and potentiating a more M2-like status to promote tumor progression, immune escape, and therapeutic resistance [2, 3, 5, 6].

Despite of the significant progress, it remains largely unknown how metabolic modes are switched at the molecular level to orchestrate the different functional statuses of macrophages at either physiological or pathogenic conditions [1, 4, 5]. In this regard, using lung cancer as a model we have demonstrated the importance of RelA (also known as p65), the prototypic member of the nuclear factor-κB (NF-κB) family of transcription factors that have been linked to almost all cancer types and inflammation-associated diseases, in both tumor biology and immunology [6–10]. Particularly, cell-intrinsic RelA not only renders TAMs resistant to the cytotoxicity of CD8^+^ cytotoxic T lymphocytes (CTLs) but also arms them with a superior capacity to suppress CTLs, thereby promoting lung cancer [6]. However, whether and how RelA regulates the metabolism and M2-like polarization of TAMs to repress anti-tumor immunity and drive tumorigenesis are yet to be determined.

**Fig. 1:**
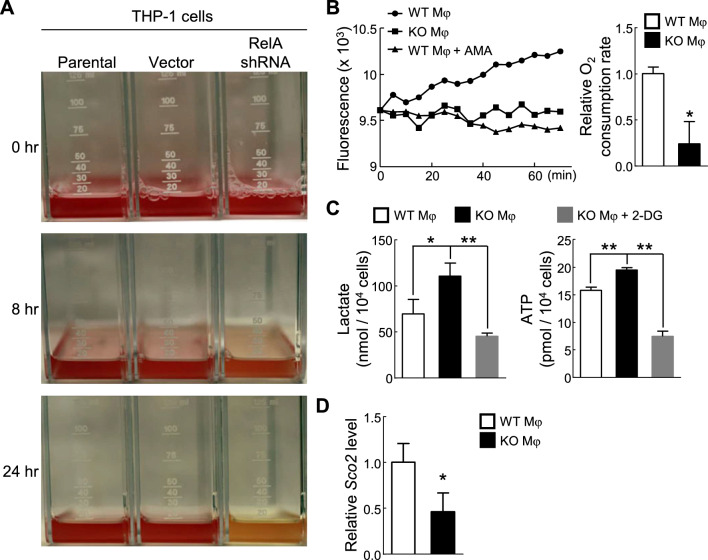
Critical role of RelA in the SCO2 expression, oxidative phosphorylation utilization and glycolysis restriction of macrophages. **A** Acidic metabolism of the monocyte/macrophage THP-1 cells by RelA knockdown. **B** Reduced oxygen consumption in RelA KO macrophages cultured in TCM. **C** Increased lactate production and ATP generation in RelA KO macrophages cultured in TCM. **D** Decreased *Sco2* expression in RelA KO macrophages cultured in TCM. In **B**–**D**, data represent means ± SEM (n = 3). * P < 0.05; ** P < 0.01; Student’s *t* test

Interestingly, RelA knockdown alone seemed sufficient to induce robust glycolysis in human monocytes/macrophages even under normal culture conditions, as indicated by the much more rapid color change to yellow of the medium culturing the THP-1 cells stably expressing RelA specific short hairpin RNAs (shRNAs) (Fig. 1A). Although THP-1 cells resemble primary monocytes and macrophages in morphology and differentiation properties, they are malignant leukaemia cells with large genomic aberrations that do not occur in primary macrophages or TAMs. To validate the promising data and more importantly to study the role RelA in the metabolism and polarization of TAMs, we examined OXPHOS and glycolytic rate in mouse primary wild type (WT) or RelA knockout (KO) macrophages cultured with the tumor-conditioned medium (TCM) of murine Lewis lung carcinoma (LLC) cells. In line with the fact that OXPHOS is the main metabolic mode of TAMs [1, 5], high oxygen consumption was detected in WT macrophages cultured in TCM (Fig. 1B). The oxygen consumption could be repressed to a basal level by the OXPHOS inhibitor antimycin A (AMA). Notably, the oxygen consumption of RelA KO macrophages cultured in the same TCM was decreased to the basal level. But in stark contrast, lactate and ATP productions were significantly increased in the same RelA KO macrophages (Fig. 1C). The increase in lactate and ATP in RelA KO macrophages could be efficiently prevented by the glycolytic inhibitor 2-deoxy-D-glucose (2-DG). Moreover, we found that synthesis of cytochrome c oxidase 2 (SCO2), a mitochondrial enzyme critical for OXPHOS, was markedly reduced in RelA KO macrophages (Fig. 1D). The SCO2 repression was specific, because no change was detected in the expression of several other metabolism-related genes, such as glucose transporter 3 (Glu3), phosphoglucomutase 2 (PGM2), ribonucleotide reductase M2 B (RRM2B) and tumor protein p53 (TP53). These data suggest that RelA induces SCO2 expression to ensure OXPHOS as the primary metabolic engine in TAMs (Additional file 1).

**Fig. 2:**
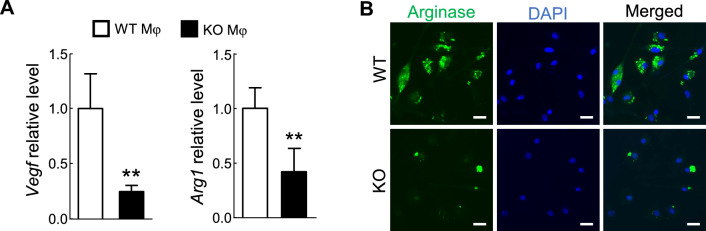
Crucial role of RelA in M2-like activation of macrophages. **A** qRCR showing decreased *Vegf* and *Arg1* in RelA KO macrophages cultured in TCM. Data represent means ± SEM (n = 3). **P < 0.01; Student’s *t* test. **B** Immunofluorescence staining showing decreased arginase-1 (ARG1, green) in RelA KO macrophages cultured in TCM. Blue: DAPI nuclear counterstain

Following the exciting results, we examined if RelA deletion affects the M2-like phenotype of TAMs. To do so, we compared the expression levels of arginase-1 and vascular endothelial growth factor (VEGF) in RelA KO and WT macrophages cultured in TCM. Arginase-1 and VEGF are the hallmarks and functional genes of M2 macrophages [1–6]. Consistent with the predominant M2-like phenotype of TAMs [1–6], WT macrophages cultured in TCM expressed high levels of arginase-1 and VEGF (Fig. 2). However, the expression levels of both genes were significantly lower in RelA KO macrophages cultured in the same TCM. Thus, in association with its role in driving metabolism toward OXPHOS, RelA pushes TAMs to an M2-like state.

Macrophages are the most abundant immune cells within lung tumor microenvironment [1–3, 5, 6] . During tumor initiation stages, they show an overall glycolytic M1 phenotype to prevent tumorigenesis. But later they become a general oxidative M2 status to promote tumor progression. Compared to the well-defined mechanisms underlying the opposite roles of M1 and M2 macrophages, the molecules governing the important switch remains largely unknown. In fact, this is a central question that needs to be addressed in the cancer and immunology fields. The data presented here suggest that RelA is a checkpoint of macrophages maintaining high OXPHOS activity to restrict glycolysis for M2-like activation and tumor pathogenesis. They thus provide new mechanistic insights into how metabolism is delicately regulated to shape the phenotype and function of macrophages and how the metabolism and function of macrophages is deregulated for pathogenesis, and in particular, cancer promotion (Additional file 2).

### Supplementary Information

**Additional file 1: **Methods.

**Additional file 2: Table S2. ** Primers used for qPCR. 

## Data Availability

All data generated or analysed during this study are included in this published article and its supplementary information files.
