# Supplementary material for: NF-κB RelA is a cell-intrinsic metabolic checkpoint restricting glycolysis
Source: Cell Biosci. 2024 Jan 20;14:11. doi: 10.1186/s13578-024-01196-7 (PMC10799406; doi:10.1186/s13578-024-01196-7)
Supplement: Supplementary file 1 — Additional file 1: Methods. [file 13578_2024_1196_MOESM1_ESM.docx]

**Additional file 1: Methods.**

**Bone marrow-derived macrophage (BMDM) generation**

Both myeloid RelA KO and WT mice were under a pure FVB/N background. Bone marrow cells were flushed from their femurs and cultured for 8 days with 10 ng/mL macrophage colony-stimulating factor (M-CSF). Non-attached cells were washed away, and adherent cells were BMDMs.

**Measurements of Lactate, ATP and Oxygen**

RelA KO and WT BMDMs were co-cultured in murine Lewis lung carcinoma (LLC) cell-conditioned medium for the indicated times. Oxygen consumption, lactate secretion and intracellular ATP levels were measured using the Oxygen Consumption Rate Assay Kit, Glycolysis Cell-Based Assay Kit (Cayman Chemical) and CellTiterGlo Reagent (Promega), according to the manufacturer’s instructions.

**Quantitative Polymerase Chain Reaction (qPCR) Analysis**

RelA KO and WT BMDMs were subjected to RNA extraction, RNA reverse transcription and qPCR. Primers for qPCR were listed in Supplementary Table S1.

**Immunofluorescence (IF) Analysis**

Cells were fixed, permeabilized, and subsequently incubated with the arginase-1 primary antibody (sc-18351, Santa Cruz Biotechnology, Dallas, TX, USA), followed by FITC-conjugated secondary antibody. Cells were also counterstained with DAPI for nuclear staining. Stained arginase-1 was detected using a fluorescence microscope.

**Statistical Analysis**

Data were reported as mean ± standard error of the mean (SEM). Student’s *t* test (two tailed) was used to assess significance of differences between two groups, and P values < 0.05 and 0.01 were considered statistically significant and highly statistically significant, respectively.
