# Supplementary material for: NF-κB RelA is a cell-intrinsic metabolic checkpoint restricting glycolysis
Source: Cell Biosci. 2024 Jan 20;14:11. doi: 10.1186/s13578-024-01196-7 (PMC10799406; doi:10.1186/s13578-024-01196-7)
Supplement: Supplementary file 2 — Additional file 2: Table S2. Primers used for qPCR. [file 13578_2024_1196_MOESM2_ESM.docx]

**Additional file 2: Table S1**

Primers used for qPCR.

| **Gene** | **Species** | **Accession number** | **Forward (5' to 3')** | **Reverse (5' to 3')** | **Usage** |
| --- | --- | --- | --- | --- | --- |
| *18S RNA* | mouse | NR_003278.3 | AGGAATTGACGGAAGGGCAC | GGACATCTAAGGGCATCACA | RT-PCR |
| *β-actin* | mouse | NM_007393.3 | ACCCGCGAGCACAGCTTCTTTG | CTTTGCACATGCCGGAGCCGTTG | RT-PCR |
| *Sco2* | mouse | NM_001111288.1 | TCATCACTGTGGACCCAGAA | TAGGCCCAGCGCTGTAGTAT | RT-PCR |
| *Vegfa* | mouse | NM_009505.4 | GCACTGGACCCTGGCTTTAC | GTCTCAATCGGACGGCAGTA | RT-PCR |
| *Arg1* | mouse | NM_007482.3 | AATCTGCATGGGCAACCTGT | GTCTACGTCTCGCAAGCCAA | RT-PCR |
